# Advisory Committee on Immunization Practices Recommended Immunization Schedule for Adults Aged 19 Years or Older — United States, 2022

**DOI:** 10.15585/mmwr.mm7107a1

**Published:** 2022-02-18

**Authors:** Neil Murthy, A. Patricia Wodi, Henry Bernstein, Veronica McNally, Sybil Cineas, Kevin Ault

**Affiliations:** ^1^Immunization Services Division, National Center for Immunization and Respiratory Diseases, CDC; ^2^Zucker School of Medicine at Hofstra/Northwell and Cohen Children’s Medical Center, New Hyde Park, New York; ^3^Fanny Strong Foundation, West Bloomfield, Michigan; ^4^The Warren Alpert Medical School of Brown University, Providence, Rhode Island; ^5^University of Kansas Medical Center, Kansas City, Kansas.

At its November 2021 meeting, the Advisory Committee on Immunization Practices* (ACIP) approved the Recommended Adult Immunization Schedule for Ages 19 Years or Older, United States, 2022. The 2022 adult immunization schedule summarizes ACIP recommendations, including several changes to the cover page, tables, and notes from the 2021 immunization schedule.^†^ In addition, the 2022 adult immunization schedule provides an appendix that lists the contraindications to and precautions for all routinely recommended vaccines in the schedule. This schedule can be found on the CDC immunization schedule website (https://www.cdc.gov/vaccines/schedules). Health care providers are advised to use the cover page, tables, notes, and appendix together. This adult immunization schedule is recommended by ACIP (https://www.cdc.gov/vaccines/acip) and approved by CDC (https://www.cdc.gov), the American College of Physicians (https://www.acponline.org), the American Academy of Family Physicians (https://www.aafp.org), the American College of Obstetricians and Gynecologists (https://www.acog.org), the American College of Nurse-Midwives (https://www.midwife.org), the American Academy of Physician Associates (https://www.aapa.org), and the Society for Healthcare Epidemiology of America (https://www.shea-online.org).

ACIP’s recommendations for the use of each vaccine are developed after in-depth reviews of vaccine-related data, including the epidemiology and societal impacts of the vaccine-preventable disease, vaccine efficacy and effectiveness, vaccine safety, quality of evidence, feasibility of program implementation, and economic analyses of immunization policy (*1*). The adult immunization schedule is published annually to consolidate and summarize updates to ACIP recommendations on vaccination of adults and to assist health care providers in implementing current ACIP recommendations. The use of vaccine trade names in this report and in the adult immunization schedule is for identification purposes only and does not imply endorsement by ACIP or CDC.

For further guidance on the use of each vaccine, including any changes that might occur between annual publication of the adult immunization schedule, health care providers are referred to the respective ACIP vaccine recommendations at https://www.cdc.gov/vaccines/hcp/acip-recs.^§^ Printable versions of the 2022 adult immunization schedule and ordering instructions are available at https://www.cdc.gov/vaccines/schedules/hcp/adult.html#note. For CDC’s interim clinical considerations for the use of COVID-19 vaccines, health care providers are referred to: https://www.cdc.gov/vaccines/covid-19/clinical-considerations/covid-19-vaccines-us.html.

## Changes in the 2022 Adult Immunization Schedule

Vaccine-specific changes in the 2022 immunization schedule for adults aged ≥19 years include new or updated ACIP recommendations for hepatitis B vaccine (HepB) (*2*), influenza vaccine (*3*), pneumococcal vaccines (*4*), recombinant zoster vaccine (RZV) (*5*), and COVID-19 vaccine (available at https://www.cdc.gov/vaccines/hcp/acip-recs/vacc-specific/covid-19.html). Changes have also been made to the human papillomavirus (HPV); measles, mumps, and rubella (MMR); meningococcal; and varicella (VAR) vaccination sections to improve clarity in the language. In addition, an appendix listing the contraindications to and precautions for each vaccine has been added to the schedule this year.

### Cover page

• A step instructing providers to review the newly added appendix has been added to the “How to use the adult immunization schedule” box.

• The Society for Healthcare Epidemiology of America has been added as a partner organization approving the adult schedule.

• PCV15 (Vaxneuvance) and PCV20 (Prevnar 20) have been added to the table of vaccine abbreviations and trade names.

• PCV13 (Prevnar 13) has been removed from the list of vaccine abbreviations and trade names.

• A QR code has been added at the bottom of the cover page for health care providers to access the online schedule (https://www.cdc.gov/vaccines/schedules/hcp/imz/adult.html).

### Table 1 (Routine Immunization Schedule)

• **Zoster row**: For adults aged 19–49 years, the color of the row was changed to purple indicating that RZV is now recommended for adults in this age group who have immunocompromising conditions. The text overlay now states, “2 doses for immunocompromising conditions (see notes).”

• **Pneumococcal row**: All recommended pneumococcal vaccines (i.e., PCV15, PCV20, and PPSV23) have been collapsed into one row. Guidance on which vaccines are indicated for certain age groups is displayed by the corresponding colors and overlying text. For adults aged 19–64 years, the row is purple, indicating that pneumococcal vaccination is recommended for adults in this age group only if they have an additional risk factor or another indication. For adults aged ≥65 years, the row is yellow, indicating that pneumococcal vaccination is universally recommended for adults in this age group, if they have never received a pneumococcal conjugate vaccine previously or if their previous pneumococcal vaccination history is unknown. The text overlay now states, “1 dose PCV15 followed by PPSV23 OR 1 dose PCV20 (see notes).”

• **Hepatitis B row**: For adults aged 19–59 years, the row is yellow, indicating that HepB vaccination is universally recommended for adults in this age group, and purple for adults aged ≥60 years, indicating that HepB vaccination is recommended for adults in this age group if they have an additional risk factor or another indication. The text overlay now states “2, 3, or 4 doses depending on vaccine or condition.”

### Table 2 (Immunization by Medical Indication Schedule)

• **Header**: For the HIV infection columns, CD4 percentages are displayed along with CD4 counts to harmonize presentation of this information with that in the child/adolescent schedule.

• **Legend**: The description of the color red in the legend has been reworded to “Contraindicated or not recommended.”

• **LAIV4 row**: The text overlay in the red box was changed to “Contraindicated” to increase clarity in the language and to align more closely with ACIP recommendations.

• **MMR row**: The text overlay for the red boxes was changed to “Contraindicated” to increase clarity in the language and to align more closely with ACIP recommendations.

• **VAR row**: The text overlay for the red boxes was changed to “Contraindicated” to increase clarity in the language and to align more closely with ACIP recommendations.

• **RZV row:** Under the Immunocompromised and HIV infection columns, the row is yellow indicating that RZV is recommended for these subgroups. In addition, the text overlay under these columns now states, “2 doses at age ≥19 years.”

• **HepB row**: The row is now entirely yellow, indicating that hepatitis B vaccination is recommended for all risk-based groups in Table 2. The text overlay states, “3 doses (see notes)” in the pregnancy column, and “2, 3, or 4 doses depending on vaccine or condition,” in the remaining columns.

### Notes

The notes for each vaccine are presented in alphabetical order. Edits have been made throughout the Notes section to harmonize language between the child/adolescent and the adult immunization schedules to the greatest extent possible.

• **COVID-19**: The hyperlinks to the ACIP recommendations for the use of COVID-19 vaccines and the CDC’s Interim Clinical Considerations for the use of COVID-19 vaccines are included in this box.

• **HepB**: The “Routine vaccination” section now states that adults aged 19–59 years are recommended to receive a 2-, 3-, or 4-dose series, with details provided. The “Special situations” section outlines the risk-based recommendations for adults aged ≥60 years. In addition, language has been added stating that “anyone age 60 years or older who does not meet risk-based recommendations may still receive Hepatitis B vaccination.”

• **HPV**: A minor edit was made to the “Routine vaccination” section to increase clarity; it now states, “No additional dose recommended when any HPV vaccine series has been completed using the recommended dosing intervals.” In addition, minor wording changes were made to the “Special situations” section, under the immunocompromising conditions sub-bullet, which now reads, “3-dose series, even for those who initiate vaccination at age 9 through 14 years.” Wording for the pregnancy sub-bullet was rearranged to improve clarity.

• **Influenza**: The language was edited to clarify the age as “19 years or older,” to be consistent with the schedule. A hyperlink to the 2021–22 influenza recommendations and a bullet for the 2022–23 influenza recommendations were added. The “Special situations” section was condensed by referring health care providers to the appendix listing the contraindications and precautions for the influenza vaccines.

• **Meningococcal vaccination**: At the end of the section, a note was added that states, “MenB vaccines may be administered simultaneously with MenACWY vaccines if indicated, but at a different anatomic site, when feasible.”

• **MMR:** In the “Special situations” section, CD4 percentages in addition to CD4 counts in the HIV infection bullet were added to harmonize language with the child/adolescent schedule.

• **Pneumococcal vaccination:** The section has been updated to reflect ACIP’s new recommendations for PCV15 and PCV20 vaccines. The “Routine vaccination” section now states that persons aged ≥65 years “who have not previously received a pneumococcal conjugate vaccine or whose previous vaccination history is unknown should receive 1 dose of PCV15 or 1 dose of PCV20. If PCV15 is used, this should be followed by a dose of PPSV23.” Similarly, the “Special situations” section has changed, and this section states that anyone “aged 19 through 64 years with certain underlying medical conditions or other risk factors who has not previously received a pneumococcal conjugate vaccine or whose previous vaccination history is unknown should receive 1 dose of PCV15 or 1 dose of PCV20. If PCV15 is used, this should be followed by a dose of PPSV23.” Guidance for dosing intervals between PCV15 and PPSV23 and for patients who have previously received PCV13 or PPSV23 in the past is also included. A note added at the end lists all the underlying medical conditions or risk factors that would render those aged 19–64 years eligible to receive pneumococcal vaccination.

• **Varicella**: In the “Special situations” section, CD4 percentages in addition to CD4 counts in the HIV infection bullet were added to harmonize language with the child/adolescent schedule.

• **Zoster**: In the “Special situations” section under the pregnancy bullet, the language was revised to increase clarity. This bullet now states, “There is currently no ACIP recommendation for RZV use in pregnancy. Consider delaying RZV until after pregnancy.” In addition, the immunocompromising conditions bullet was revised to reflect the new ACIP recommendations for zoster vaccination. This bullet now states, “RZV is recommended for use in persons aged 19 years and older who are or will be immunodeficient or immunosuppressed because of disease or therapy.”

### Appendix (Contraindications and Precautions)

• The appendix includes all the contraindications to and precautions for each of the vaccines listed in the 2022 adult immunization schedule. The information presented in this appendix is adapted from the 2021–22 influenza vaccine recommendations (*3*) and from ACIP General Best Practice Guidelines for Immunization (*6*).

## Additional Information

The Recommended Adult Immunization Schedule, United States, 2022, is available at https://www.cdc.gov/vaccines/schedules/hcp/adult.html and in the *Annals of Internal Medicine *(https://www.acpjournals.org/doi/10.7326/M22-0036). The full ACIP recommendations for each vaccine are also available at https://www.cdc.gov/vaccines/hcp/acip-recs/index.html. All vaccines identified in Tables 1 and 2 (except PCV15, PCV20, and zoster vaccine) also appear in the Recommended Immunization Schedule for Children and Adolescents, United States, 2022 (https://www.cdc.gov/vaccines/schedules/hcp/imz/child-adolescent.html). The notes and appendices for vaccines that appear in both the adult immunization schedule and the child and adolescent immunization schedule have been harmonized to the greatest extent possible.

## Footnotes

* Recommendations for routine use of vaccines in adults are developed by ACIP, a federal advisory committee chartered to provide expert external advice and guidance to the CDC director on use of vaccines and related agents for the control of vaccine-preventable diseases in the civilian population of the United States. Recommendations for routine use of vaccines in adults are harmonized to the greatest extent possible with recommendations made by the American Academy of Pediatrics, the American Academy of Family Physicians, and the American College of Obstetricians and Gynecologists. ACIP recommendations approved by the CDC director become agency guidelines on the date published in the *Morbidity and Mortality Weekly Report*. Additional information about ACIP is available at https://www.cdc.gov/vaccines/acip.

† Past immunization schedules are available at https://www.cdc.gov/vaccines/schedules/past.html.

§ CDC encourages organizations to use syndication as a more reliable method for displaying the most current and accurate immunization schedules on an organization's website rather than copying these schedules to their websites. Use of content syndication requires a one-time step that ensures an organization’s website displays current schedules as soon as they are published or revised; instructions for the syndication code are available on CDC’s website (https://www.cdc.gov/vaccines/schedules/syndicate.html). CDC also offers technical assistance for implementing this form of content syndication (requests can be e-mailed to ncirdwebteam@cdc.gov).
